# How Phenograms and Cladograms Became Molecular Phylogenetic Trees

**DOI:** 10.1007/s10739-024-09782-8

**Published:** 2024-08-30

**Authors:** Nina Kranke

**Affiliations:** Chair of Nature Conservation and Landscape Ecology, University of Freiburg, Freiburg, Germany

**Keywords:** Phenogram, Cladogram, Phylogenetic tree, Tree diagram, Systematics, Molecular evolution

## Abstract

Tree diagrams are the prevailing form of visualization in biological classification and phylogenetics. Already during the time of the so-called Systematist Wars from the mid-1960s until the 1980s most journal articles and textbooks published by systematists contained tree diagrams. Although this episode of systematics is well studied by historians and philosophers of biology, most analyses prioritize scientific theories over practices and tend to emphasize conflicting theoretical assumptions. In this article, I offer an alternative perspective by viewing the conflict through the lens of representational practices with a case study on tree diagrams that were used by numerical taxonomists (phenograms) and cladists (cladograms). I argue that the current state of molecular phylogenetics should not be interpreted as the result of a competition of views within systematics. Instead, molecular phylogenetics arose independently of systematics and elements of cladistics and phenetics were integrated into the framework of molecular phylogenetics, facilitated by the compatibility of phenetic and cladistic practices with the quantitative approach of molecular phylogenetics. My study suggests that this episode of scientific change is more complex than common narratives of battles and winners or conflicts and compromises. Today, cladograms are still used and interpreted as specific types of molecular phylogenetic trees. While phenograms and cladograms represented different forms of knowledge during the time of the Systematist Wars, today they are both used to represent evolutionary relationships. This indicates that diagrams are versatile elements of scientific practice that can change their meaning, depending on the context of use within theoretical frameworks.

## Introduction

During the time from the 1960s until the 1980s, systematics was characterized by heated and often emotional debates over different approaches to biological classification and taxonomic practices. This episode in the history of biology is often referred to as “Systematist Wars” (Hull 1988).^1^ The three main contestants in the conflict were evolutionary systematists, numerical taxonomists, and cladists. Most of the research on the conflict in theoretical biology as well as history and philosophy of biology prioritizes scientific theories over practices and tends to emphasize conceptual differences between the opposing parties, e.g. about biological units, speciation, and classification. A prominent example is David Hull’s (1988) account of the competing theories as independent historical lineages of ideas. Brower and Schuh (2021) also characterize evolutionary systematics, phenetics, and cladistics as different “schools” of systematics separated by a “philosophical gulf.”^2^ According to Hull’s (1988) account cladists eventually won the war while Brower and Schuh describe the end of the conflict as a “consensus […] that the cladistic approach offers compelling methodological and philosophical advantages over those alternatives” (Brower and Schuh 2021, pp. 19–20). In both narratives cladistics is portrayed as the school of systematics that has prevailed, but both accounts focus only on the history of systematics and largely ignore other important developments that have led to the rise of molecular phylogenetics. More recent accounts challenge the view of scientific theories as abstract conceptual systems and propose a practice-oriented approach to studying this episode in systematics.^3^ Following this practice-oriented view I examine numerical taxonomy and cladistics as two different approaches of doing systematics by analyzing their representational practices.

In the sciences, particularly in the biological sciences, visualization plays a pivotal role, at times to the extent that text illustrates images, not the other way around. Diagrams are used to graphically communicate scientific results and hypotheses to fellow scientists and to a broader public. As research aims at producing knowledge of a certain type, often represented in specific formats, diagrams structure and guide scientific research. The analysis of representational practices thus sheds light on central topics in history and philosophy of science such as processes of scientific change and continuity of practices.

Both pheneticists and cladists have used tree diagrams to visually represent their results. The “iconographic tradition” (Gould 1995) of using tree-shaped images and diagrams to represent relationships between individual organisms or groups of organisms started long before Darwin published his famous branching diagram in *On the Origin of Species* in 1859 (Ragan 2009). Tree images and diagrams have developed into “canonical icons” in biology, particularly in evolutionary biology and systematics (Gould 1995). Today, phylogenetic trees are essential tools for studies in evolutionary biology, but before the period of the Systematist Wars tree diagrams were first and foremost used for classification.

In this article I describe the developments that have led to the rise of molecular phylogenetics and argue that this field did not arise from systematics, but from a different disciplinary context. This is important to understand that the conflicts in systematics that revolved around biological classification were overshadowed by ongoing processes of evolutionization, mathematization, automation, and quantification. I argue that the construction of phylogenetic trees by using statistical methods was initiated independently in systematics and molecular evolution. These practices were integrated and further developed to eventually dominate molecular phylogenetics. In this process phenograms were reinterpreted as molecular phylogenetic trees and cladograms became molecular phylogenetic trees that do not represent the amount of evolutionary change within lineages. With the integration of practices of systematics with molecular evolution, phenograms and cladograms are no longer used to represent different forms of knowledge and became different kinds of phylogenetic trees in the context of molecular phylogenetics.

## Conflicts in Systematics

By the time the so-called Systematist Wars started, the established approach to biological classification was evolutionary taxonomy, also called *evolutionary systematics*, previously called the *new systematics* (Sterner and Lidgard 2018). The most well-known proponents of evolutionary systematics were the zoologists Ernst Mayr and George G. Simpson. Classification based on evolutionary taxonomy emphasized the importance of evolution and speciation processes (Mayr 1969; Simpson 1961). According to Mayr’s biological species concept, species are interbreeding populations that are reproductively isolated from other populations caused by a period of geographic isolation (Mayr 1942).^4^ To study the degree of divergence between groups of organisms, evolutionary systematists evaluated morphological characters across geographic ranges of populations. The construction of evolutionary trees as a basis for classification involved the weighting of characters and formation of groups based on previously established phylogenetic hypotheses. With the rise of numerical taxonomy and cladistics, the established approach was challenged to its methodological foundations by proponents of these alternative approaches (Hull 1988; Suárez-Díaz and Anaya-Muñoz 2008).

Phenetic approaches to classification emerged in the late 1950s as an important part of a broader approach of implementing numerical methods in biological systematics called *numerical taxonomy*. Due to the influence of their book *Principles of Numerical Taxonomy* published in 1963, microbiologist Peter Sneath and statistician Robert Sokal are portrayed as the main advocates of phenetic classification (Sokal and Sneath 1963). In phenetics statistical methods are applied to generate clusters of similar organisms based on overall similarity. To create a hierarchical classification, the clusters can be joined together and form higher level units. Thus, the phenetic classification approach does not require phylogenetic analysis or reference to speciation processes. Instead, classification and phylogenetic inference, the two main tasks of systematics, are treated as separate and independent from each other. In fact, part of the broader program pursued by numerical taxonomists was to perform cladistic analysis by applying numerical methods, or numerical cladistics (Sneath and Sokal 1973). Sneath and Sokal explicitly emphasized that numerical taxonomy “includes the drawing of phylogenetic inferences from the data by statistical or other mathematical methods” (Sneath and Sokal 1973, p. 4).

Evolutionary systematics and phenetics were challenged by cladistics, also called *phylogenetic systematics*. The cladistic approach goes back to the entomologist Willi Hennig and is based on the recognition of monophyletic groups or clades, defined as “a group of species descended from a single (‘stem’) species, and which includes all species descended from this stem species” (Hennig 1966, p. 73). Monophyletic groups can be identified by shared derived characters. According to cladists classification should reflect phylogenetic relationships. My analysis of phenograms and cladograms in the following section shows the similarities and differences between practices in cladistics and phenetics in more detail.

## Representing Results in Phenetics and Cladistics

To understand the differences and similarities between phenetics and cladistics I examine two exemplary diagrams, a phenogram and a cladogram, by analyzing their components, graphic structures, meanings, as well as the context of their construction and use. As exemplars, these diagrams represent common features of most phenograms and cladograms that were used during the time period in question. The phenogram was published by Gary Schnell in *Systematic Zoology* in 1970 and the cladogram was published by Greg Spicer in the *Journal of Crustacean Biology* in 1985.

**Fig. 1:**
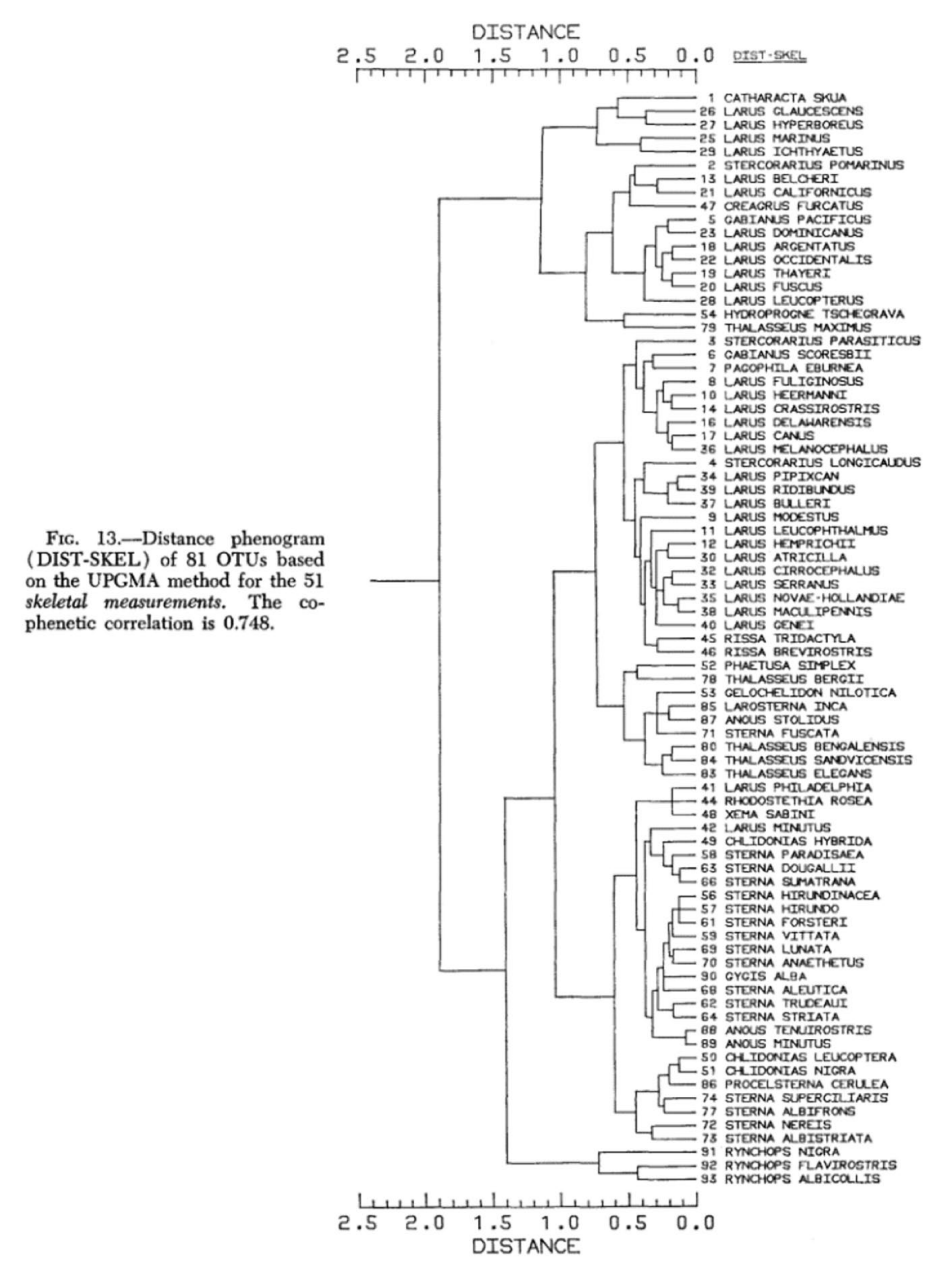
Phenogram with original caption (Schnell 1970)

**Fig. 2:**
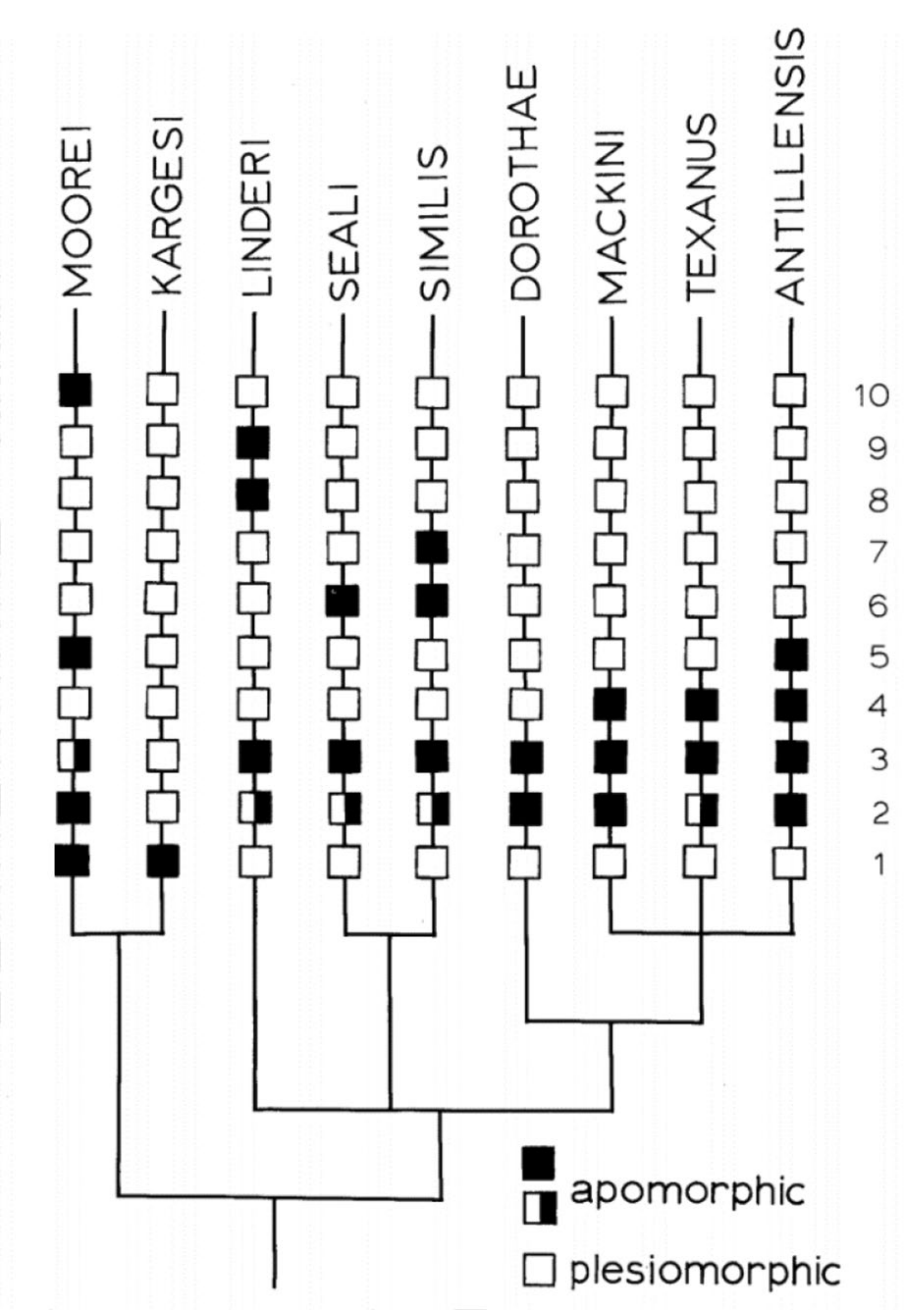
Cladogram with original caption (Spicer 1985)

Already at a first glance, it becomes clear that both diagrams share basic components and have structural similarities. Both the phenogram (Fig. 1) and the cladogram (Fig. 2) are composed of vertical and horizontal lines that form a branching structure with a predominantly bifurcating pattern. However, the phenogram’s root is on the left and the tips of the branches are on the right, whereas the cladogram’s branches are organized from bottom to top. This depiction of the phenogram on its side has a pragmatic reason. Sneath and Sokal explained, “[a]lthough early practice tended to have the branches of a phenogram pointing upwards, convenience and the ever increasing size of studies have made authors place phenograms almost uniformly on their side with branches running horizontal across the page” (Sneath and Sokal 1973, p. 260).

This statement generally also holds true for cladograms, but as the number of species represented in the cladogram by Spicer is relatively small, the branches run from bottom to top (Fig. 2). In addition to the schematic tokens, both diagrams contain words and numbers with a similar degree of pictorial abstraction, meaning that both diagrams are highly schematized trees as opposed to more figurative tree diagrams like Ernst Haeckel’s famous oak tree. Each diagram also has unique components. The cladogram has three different types of square-shaped symbols and a key that indicates the meaning of the symbols, and the phenogram has a labelled x-axis. Although many alternative diagrammatic forms of representation existed in numerical taxonomy, e.g., ordination plots, contour diagrams,^5^ pheneticists often used tree diagrams to represent their results because hierarchical classification systems could easily be derived from them. Sneath and Sokal claimed that “[t]he results of cluster analysis have been traditionally represented by dendrograms [i.e. tree diagrams], which have the advantage that they are readily interpretable as conventional taxonomic hierarchies” (Sneath and Sokal 1973, p. 260). Tree diagrams were used due to mathematical conventions, but they were also preferred because of their purpose as classification tools. In cladistics, however, tree diagrams were the only form of diagrammatic representation.

## What Phenograms Represent and How They Are Constructed

As already mentioned, phenetics is an approach to taxonomy that classified organisms based on resemblance, and phenograms were used to represent the degree of similarity between groups of organisms, so called “phenetic relationship” (Sneath and Sokal 1973, p. 29).^6^ In the phenogram by Schnell (Fig. 1) the numbers represent extant OTUs, or Operational Taxonomic Units, also referred to as “phenons,” and the words next to the numbers are species names and represent previously identified and named species. Pheneticists insisted that although phenons can be equated with rank categories such as “species,” they are not fully synonymous with taxa. It was very important to pheneticists to avoid the evolutionary connotations of established terms like “taxa” or “species” to emphasize the difference between their approach and competing approaches to classification (Sneath and Sokal 1973). It is important to understand that phenograms had not been used to represent evolutionary relationships of any kind,^7^ only degrees of similarity indicated by the position of the nodes in the diagram. In this phenogram, for example, OTUs 26 and 27 share more similarities with each other than with OTU 1 (Fig. 1, top of the diagram).

Phenograms were constructed by means of numerical methods using phenotypic characters. According to Sneath and Sokal, “[w]hat one wishes to measure in phenetic taxonomy is the expression of the genome of the organism through its life history– its phenome, in fact” (Sneath and Sokal 1973, p. 96). Usually, a large number of characters were used to generate phenograms. To construct the phenogram by Schnell, 51 skeletal measurements of gulls were analyzed applying Unweighted Pair Group Method with Arithmetic Mean, or UPGMA cluster analysis, a statistical method for evaluating relationships (Schnell 1970; see caption in Fig. 1). As it was tedious to do phenetic clustering and other numerical methods by hand, the introduction of computers into systematics research in the 1960s had simplified and accelerated the pheneticists’ work enormously (Hagen 2001). However, by the time Schnell conducted his study, cheap personal computers were not yet available and researchers had to rely on computation facilities. In his acknowledgements Schnell explicitly expresses his gratitude for the “computer time [that] was made available by the Computation Center at the University of Kansas” (Schnell 1970, p. 301).

## What Cladograms Represent and How They Are Constructed

Cladograms are diagrams that were used for representing patterns as results of evolutionary processes, not simply similarities. While phenograms represented similarities between organisms, cladograms represented kinship relationships. During the times of the Systematist Wars there were controversial discussions focusing on what cladograms represent and in what respect they differ from phylogenetic trees. Until the mid-1970s cladograms were usually understood as phylogenetic trees justified by synapomorphic characters (Wiley 1981). In the following years a debate flared up as a reaction to a widely circulated, but never published manuscript by Gareth Nelson. In this paper, he stated that cladograms are not phylogenetic trees but tree diagrams representing patterns of unique characters (Eldredge and Cracraft 1980; Wiley 1981). Following his understanding of cladograms, Eldredge and Cracraft argued, that “a cladogram subsumes the logical structure of a set of trees. Phylogenetic trees, in specifying actual series of ancestral and descendant taxa, are more detailed and precise sorts of hypotheses than are cladograms” (Eldredge and Cracraft 1980, p. 10). From this perspective, cladograms are tree diagrams without specified ancestors. The distinction of cladograms and phylogenetic trees resulted in the common opinion that a large array of phylogenetic trees existed for each cladogram.^8^ Cladists like Eldredge and Cracraft understood cladograms “as diagrams of the history of taxa [which] can be interpreted in terms of relative recency of common ancestry” (Eldredge and Cracraft 1980, p. 10).^9^

Since cladograms were constructed from characters classified as evolutionary novelties and ancestors do not exhibit novelties unique to themselves, it is difficult to make justified claims about ancestors. This argument had led cladists to focus on identifying nested sets of unique characters depicted by branching diagrams (Eldredge and Cracraft 1980). The interpretation of cladograms as diagrams exhibiting patterns of character distributions did not require additional hypotheses about speciation events or specific assumptions about evolutionary processes. Due to this interpretation cladograms were considered a suitable basis for classification. Eldredge and Cracraft claimed that “[t]he procedure has the added advantage of being easily converted into classifications with a minimum of required conventions” (Eldredge and Cracraft 1980, p. 10). The emphasis on character distributions is obvious in the exemplary cladogram in Fig. 2 where the numbers 1–10 represent different characters and the different square symbols indicate whether the character or character state in question is apomorphic or plesiomorphic in the respective species.^10^ As in the phenogram, the words at the tips of the branches are species names and represent extant species. The diagram by Spicer in Fig. 2 can be interpreted as follows. *S. moorei* is more closely related to *S. kargesi* than to the other species in this group. In other words, *S. moorei* is the sister group or sister species of *S. kargesi*. No information about actual or hypothetical common ancestors is given in the cladogram or in Spicer’s article.

Both phenograms and cladograms were based on shared characters, which is why “phenetic similarity may be an indicator of cladistic relationship,” but “it is not necessarily congruent with the latter” (Sneath and Sokal 1973, p. 29). This discrepancy between phenetic similarity and cladistic relationships was caused by the cladists’ interpretation of characters as ancestral or derived. In the cladogram shown in Fig. 2, S. *moorei* and *S. kargesi* both have a rounded frontal appendage, character 1, that is unique for this group and therefore an apomorphic character (Spicer 1985). In the other 7 species represented in the diagram, the frontal appendage is bilobed, a character state not unique to this subgroup, because it was inherited from the ancestor they share with *S. moorei* and *S. kargesi*. Thus, rounded frontal appendages are understood as an evolutionary novelty. Pheneticists, on the other hand, did not differentiate between different types of character states and base their analysis solely on *unweighted* similarity.

As it is not possible to gain direct knowledge of historical patterns, cladists used parsimony algorithms to infer evolutionary relationships.^11^ This means that of all possible cladograms for the group in question, the cladogram that minimizes the total number of character state changes was to be preferred. The cladogram in Fig. 2 is a so-called *Wagner Tree* and was constructed with the aid of a computer program called *Wagner-78* that applied parsimony to cladistic analysis.^12^ Spicer used 10 morphological characters of shrimp species, e.g. teeth, fingers, and spines, to construct his cladogram.

## Similar Practices with Different Ontologies

The analysis of the phenogram and the cladogram and the corresponding practices show that pheneticists and cladists were committed to different ontologies in the sense that they had different ways of grouping. Pheneticists classified operational taxonomic units into groups of phenons, whereas cladists followed the Linnaean classificatory system that classifies groups of organisms into taxa such as species and genera. For pheneticists like Sneath it was important that “the ‘natural’ classification would contain the most information, be highly predictive and would have the most general purpose” (Vernon 1988, p. 149). To achieve this goal, pheneticists based their analysis on many unweighted characters, so that the classification system would reflect different degrees of overall similarity. From a cladist’s point of view, on the other hand, a “natural” classification system should reflect evolutionary relationships. Thus, the controversy between cladists and pheneticists revolved around the question whether or not a classification should represent evolutionary relatedness.

Despite these fundamental disagreements, phenetic and cladistic practices also had a number of similarities as illustrated by this case study. Both schools used tree diagrams as a basis for classification and both diagrams were constructed by the use of computers based on mathematical algorithms and morphological characters. The fact that both parties were striving for objective classifications by avoiding human intervention and judgment as far as possible suggests a shared ideal of scientific objectivity.^13^ I will argue that the compatibility of these practices with practices in molecular evolution and the ideal of objectivity that evolved in systematics within the 20th century enabled the integration of elements from systematics into molecular phylogenetics. In the following two sections I will give an outline of the developments in systematics and molecular evolution that facilitated this integration.

## 20th Century Taxonomy: Evolutionization, Mathematization and Automation

In the late 19th and early 20th century, taxonomy suffered severe image problems to the extent that it was considered old-fashioned, out-of-date, and unscientific and became increasingly unattractive not only to funding bodies, but also to other biologists and biology students (Vernon 1993). Taxonomy was considered an outdated discipline mainly because the methods and practices had not significantly changed with the acceptance of Darwin’s theory of evolution and natural selection. Morphology-based classifications were simply reinterpreted in evolutionary terms based on the assumption that established methods produced “natural” taxa which could readily be interpreted phylogenetically. To update the discipline and make it more explicitly scientific, new methods, data, technologies, and theories, in short, new ways of practicing taxonomy were introduced in the course of the 20th century (Hagen 2001).^14^

In order to replace the notion of “old taxonomy,” Julian S. Huxley (1940) coined the term *new systematics* that was used to summarize the early attempts to revamp and evolutionize the discipline by including evidence from cytology, ecological data, as well as considering geographic variation and reproductive relationships (Vernon 1993). In the 1940s and 1950s, Mayr and Simpson, two of the main architects of the evolutionary synthesis, initiated a new approach of practicing systematics by emphasizing the connections between taxonomic and evolutionary work. To make evolutionary aspects explicit and more central, they focused on speciation and reproductive behavior and introduced paleontological data, studies of populations in the field, breeding experiments, physiological evidence, and evidence from genetics and embryology to taxonomic practice. Their approach, today known as *evolutionary systematics*, contributed substantially to firmly rooting mainstream taxonomic practices in evolutionary theory (Vernon 1993).

In the late 1950s numerical taxonomy arose as a competing approach. The main reason for developing new ideas was a general dissatisfaction with the current state of taxonomy, particularly with its evolutionary foundation that was seen as a source of speculation (Vernon 1988). The ideas that gave rise to this new school of practicing taxonomy were formulated independently by three different groups, namely Arthur J. Cain and Geoffrey A. Harrison, Robert R. Sokal and Charles D. Michener, and Peter H. A. Sneath (Vernon 1988). Although there were great differences between the groups, they agreed on central features such as the separation of classification and phylogenetic reconstruction (Vernon 1988). Their quantitative approach slightly predated the introduction of computers into systematics. Although the origin of numerical taxonomy was not a consequence of technological advances, computers played an important role in the subsequent development of a quantitative formalism (Sterner and Lidgard 2014).^15^ Although numerical taxonomists advocated a non-evolutionary classification system, they introduced numerical approaches of cladistic analysis (Camin and Sokal 1965). This shows that numerical taxonomy was a broader program not limited to phenetic classification, but to numerical taxonomists phylogenetic inference and classification were two separate activities.

While Mayr advocated a qualitative approach based on expert judgment, numerical taxonomists suggested a quantitative approach based on automated procedures (Sterner and Lidgard 2014). These two approaches were based on different ideals of scientific objectivity. Both Mayr and Simpson valued expert knowledge and experience, whereas Sneath and Sokal tried to avoid human judgment which, to them, was the source of subjectivity (Hagen 2001). They regarded computers and automated mathematical procedures as more reliable than trained judgment because the results produced by automated procedures were reproducible with different taxonomists independently arriving at the same classification scheme (Hagen 2001). In the eyes of numerical taxonomists, therefore, the weighting of characters as practiced by evolutionary taxonomists and their idiosyncratic methods did not lead to objective classifications (Suárez-Díaz and Anaya-Muñoz 2008).

In the mid-1960s cladistic approaches emerged, largely associated with Willi Hennig. Cladists argued that classifications should reflect evolutionary history which could be achieved through the identification of monophyletic groups. They also introduced new practices to systematics, e.g., the inference of phylogenetic relationships by applying the parsimony principle. Hennig’s work did not explicitly suggest a mathematical approach, but due to its emphasis on explicit rules and formal logic, cladistic analysis was suitable for computer programming. According to Hagen (2001), parsimony was appealing to systematists because it could be explicitly defined in mathematical terms, even though the application of parsimony algorithms was questionable on biological and philosophical grounds. Already in the late 1960s numerical methods of phylogenetic inference based on Hennig’s theory were developed (Farris et al. 1970).^16^

Both cladists and numerical taxonomists had developed numerical methods for cladistic analysis and further analysis of phenetic and cladistic approaches revealed “shared elements in the computational workflows of phenetic and cladistic theories” (Sterner and Lidgard 2018, p. 54). Sterner and Lidgard’s analysis of workflows and methodologies in systematics suggests that “systematists made methodological progress in ways that depended on positive sharing of ideas between otherwise polarized social groups” (Sterner and Lidgard 2018, p. 54). Both cladists and numerical taxonomists promoted the interlinked processes of mathematization and automation within their own theoretical frameworks and due to shared practices, they were able to borrow ideas from each other. Eventually, the ongoing automation of systematics undermined the informal judgements of evolutionary taxonomy (Hagen 2001).

This short overview shows that the attempts of scientization and formalization of taxonomy gave rise to different theoretical frameworks of doing systematics. However, it also shows similarities between the opposing parties. Both evolutionary systematists and cladists argued that classifications should reflect evolutionary relationships, and cladists as well as numerical taxonomists both used numerical methods to create classifications. These similarities are also expressed in the shared representational practices discussed in the previous section. It becomes clear that systematics underwent a shift of emphasis from classification and other related activities such as describing and naming of species to studies of evolutionary relationships. During the 20th century the interrelated processes of mathematization, automation and evolutionization were initiated. The further development of these processes set the stage for the introduction and eventual dominance of molecular characters into systematics. I argue that the similarities between phenetics, cladistics and molecular phylogenetics eventually made the conversion of phenograms and cladograms into molecular phylogenetic trees possible. First, however, I will give an outline of some parallel, yet independent, developments in molecular evolution that gave rise to molecular phylogenetics.

## The Rise of Molecular Phylogenetics

The use of molecular data, broadly defined as including molecules and molecular reactions, to study relationships among species has a long history that started in the late 19th century.^17^ In this section I focus on developments in the field of molecular evolution that emerged in the 1960s at the interface of molecular biology, biochemistry, evolutionary biology, biophysics and studies on the origin of life, and exobiology (Suárez-Díaz 2009). Since the time of its origination, the field was characterized by an ongoing process of quantification and automation. However, in the 1960s, sequencing a complete protein was a time-consuming and difficult procedure, so that protein sequences could not yet be used for quantitative analysis (Suárez-Díaz 2014). The first fully automated sequencing machine, called “sequenator,” was developed by Pehr Edman in the late 1960s (García-Sancho 2012). As sequencing automation had started with proteins, it is not surprising that the first computer-generated phylogenetic trees were also based on protein structure (Hagen 2001).^18^

Molecular evolutionists who followed a comparative approach were interested in using molecular characters to study relationships among species and reconstruct phylogenetic relationships. Emile Zuckerkandl and Linus Pauling, who introduced the concept of the molecular clock at a conference in 1964, belonged to this group of researchers. Biochemists Emanuel Margoliash and Walter Fitch shared this comparative approach. Mainly because of his important contribution to developing algorithms for the inference of molecular phylogenies, Fitch is considered the founder of molecular phylogenetics (Atchley 2011). Fitch and Margoliash published their computer-generated molecular phylogenetic tree in 1967. However, the first computer-generated molecular phylogenetic tree was published by physical chemist Margaret Dayhoff and mathematician Richard Eck in 1966 (Hagen 2001). Two equally important pioneers in the field of molecular phylogenetics, population geneticist L. L. Cavalli-Sforza and statistician A. W. F. Edwards, constructed the first computer-generated molecular phylogenetic tree for human populations published in 1967 (Cavalli-Sforza and Edwards 1967). These researchers’ primary interest was the study of molecular evolution and none of them had been trained in taxonomy, so they were not particularly concerned with theories of biological classification.^19^ In the early days of molecular evolution, most of these researchers were not aware of the ongoing conflicts between proponents of numerical taxonomy, evolutionary taxonomy, and cladistics. Their computational approaches therefore did not map neatly onto one of the different schools of systematics (Hagen 2001). Cavalli-Sforza and Edwards, however, explicitly discuss the relation of their work to taxonomy, particularly to numerical taxonomy. They state, “[a]lthough data suitable for our type of evolutionary study may seem to be largely taxonomic, it should be noted that the aim of this work is not the same as that of taxonomy, as the word is normally understood […]; in particular, ‘numerical taxonomy’ […] is not primarily concerned with phylogeny, and the fact that the techniques to be described here and those of numerical taxonomy both involve the treatment of ‘taxonomic’ data should not be allowed to mask the differences between them, either at the logical or methodological levels” (Cavalli-Sforza and Edwards 1967, pp. 550–551).^20^

Although both numerical taxonomists and molecular evolutionists followed a quantitative approach, Cavalli-Sforza and Edwards clearly distanced themselves from the theoretical foundations of numerical taxonomy. Interestingly, but not surprisingly, Sneath and Sokal (1973) embraced the new approaches from molecular evolution and presented them as if they were part of the broader program of numerical taxonomy. In their chapter “Numerical Approaches to Cladistic Analysis” the approaches by Edwards and Cavalli-Sforza, Camin and Sokal, Farris and his collaborators, Fitch and Margoliash and Dayhoff are all mentioned in the first paragraph. This way of presenting their research does not clarify the underlying conceptual differences and leaves the reader with the impression that these are simply different methods of numerical taxonomy. It is true, that “[m]athematically, the computational approaches used by molecular evolutionists could be considered extensions of numerical taxonomy” (Hagen 2001, p. 303), but this description ignores the different disciplinary contexts of their origin. The distinctions between systematics and molecular evolution became even more blurred when molecular data began to dominate phylogenetic analysis in the 1980s.^21^ In molecular evolution sequences started to dominate over experimental techniques due to technological advancements, particularly the automation of sequencing (Suárez-Díaz 2014). Only then it was possible to generate a sufficient amount of digitized data for sophisticated statistical analysis.

This brief overview of the history of molecular evolution shows that the field did not arise from systematics, but in a different disciplinary context. However, systematists rapidly adopted the computational approaches used by molecular evolutionists. Eventually, sequences also prevailed in systematics because molecular data were considered “cleaner” and provided more direct evidence of evolution than morphological data. Furthermore, sequences were particularly suitable for quantitative analysis due to their discrete nature and they could be used for comparative studies between all species including prokaryotes (Suárez-Díaz and Anaya-Muñoz 2008).

The seamless integration of molecular computational approaches into systematics was only possible because systematists and molecular evolutionists had overlapping interests such as studying phylogenetic relationships, and because both fields were characterized by ongoing processes of mathematization, automation, and quantification. The introduction of computing was not solely responsible for this; the establishment of databases for molecular sequences was also a contributing factor as was the improvement of automatic sequencing.^22^ Today, molecular phylogenetics is situated at the intersection of molecular evolution and systematics. In the next section I will discuss how methods and representational practices that originated in systematics were integrated into molecular approaches of phylogenetic inference.

## How Phenograms and Cladograms Became Molecular Phylogenetic Trees

In this section I will focus on the context and agent dependent aspects of diagram use and interpretation to argue that phenograms and cladograms were reinterpreted in the context of molecular phylogenetics and are now understood as tree diagrams that represent evolutionary relationships.

Marion Vorms (2011) builds on Nelson Goodman’s (1976) notion of a symbol system to analyze the relationship between a model and its user, but his notion of a symbol system and his distinction between syntactic and semantic properties are also applicable to other types of representational systems like diagrammatic symbol systems. In order to extract information from a diagram, the user needs knowledge of the system’s syntax and semantics. Thus, before the user is able to make inferences from the diagram to features of its target, they need to know how to read the diagram. Particularly in scientific contexts, drawing information from a diagram can require a substantial amount of background knowledge (Vorms 2011). Vorms argues that “[f]or a given graph, the system that defines it determines which of its features are syntactically relevant, and how they are to be interpreted” (Vorms 2011, p. 260). Following this line of argument, she shows that format and cognitive accessibility of models or diagrams are agent and context dependent. I will apply Vorms’ approach to discuss the integration of cladograms and phenograms into the new context of molecular phylogenetics, which can be understood as a shift into a new representational system.

As tree diagrams the two diagrams presented above have the same basic format and are used in the same broad context of biological systematics. The components of the diagrams are very similar due to mathematical and inner-disciplinary conventions and they were used for a similar purpose, namely classification. Without the additional information that the tree diagram in Fig. 1 is a phenogram, it could easily be mistaken for an evolutionary tree. Already in the 1960s, Hennig pointed out that using the same format to represent different forms of knowledge could cause confusion.^23^ We can only understand that phenograms and cladograms result from applying different methods, different reasoning processes and approaches in two distinct communities by carving out the agent and context dependency of diagram construction and interpretation. Diagrams not only represent aspects or components of the world, but also theories, interests, concepts, and beliefs of individual researchers or an entire scientific community. On the one hand this means that knowledge of the context of a diagram’s construction and use is important to correctly interpret the diagram. One the other hand, some aspects of the underlying theories or beliefs etc. become visible in the diagrams through symbols, numbers, and words. One example is the labeling of apomorphic and plesiomorphic characters in the cladogram by Spicer (Fig. 2) that represent aspects of evolutionary theory. In the phenogram by Schnell (Fig. 1) the quantitative approach is visible in the number of characters and OTUs.

In molecular phylogenetics, results are still represented with tree diagrams. Already at a first glance at the contents of recent molecular phylogenetics textbooks, it becomes clear that UPGMA and parsimony are considered valid methods for phylogeny reconstruction.^24^ In the case of parsimony, this might not seem particularly surprising, but one might ask how a phenetic clustering method ended up in phylogenetics textbooks.

During the Systematist Wars pheneticists and cladists agreed that phenograms constructed with clustering algorithms such as UPGMA represent phenetic similarity, not evolutionary relationships. Nowadays, however, tree diagrams constructed with clustering algorithms are used to represent phylogenetic relationships. In *The Phylogenetic Handbook* Anne-Mieke Vandamme states, “[UPGMA] is probably the oldest and simplest method used for constructing phylogenetic trees from distance data” (Vandamme 2009, p. 26). This statement shows that the construction method and the representational format have not changed, only the interpretation of the diagram. The shift can only be explained with reference to the context of use. The following statement from a textbook illustrates the reinterpretation of phenograms as molecular phylogenetic trees:

[t]his method [i.e., UPGMA] is often attributed to Sokal and Michener (1958), but the method used by these authors is quite different from the currently used version. Its clear-cut algorithm appears in Sneath and Sokal’s (1973) book. A tree constructed by this method is sometimes called a phenogram, because it was originally used to represent the extent of phenotypic similarity for a group of species in numerical taxonomy. *However*,* it can be used for constructing molecular phylogenies when the rate of gene substitution is more or less constant*. (Nei and Kumar 2000, p. 87, emphasis added)

Thus, the interpretation of phenograms is modified in accordance with evolutionary theory by adding the criterion of constant substitution rates which goes back to Zuckerkandl and Pauling’s concept of the molecular clock (Van de Peer and Salemi 2009). This process of evolutionization took place within the context of a shift in emphasis from classification to phylogenetic inference in systematics. The transformation of phenograms into molecular phylogenetic trees was possible because UPGMA as a statistical method fit well into the quantitative framework of molecular phylogenetics and morphological characters could easily be replaced with molecular characters without the need of changing the algorithm. As already mentioned in the previous section, the approaches of numerical taxonomy and molecular evolution were mathematically similar. The context and agent dependency of interpreting the results of cluster analysis was already emphasized by Sneath and Sokal. They note, “[m]ost similarity coefficients and clustering algorithms employed in numerical cladistics are also employed in numerical phenetics. The important distinction between phenetic and cladistic analysis lies not in the similarity coefficients or clustering algorithms, therefore, *but in the assumptions underlying their use* in numerical cladistics *and in the conclusions drawn from the results of the study*” (Sneath and Sokal 1973, pp. 323–324, emphasis added). Most authors, however, are aware of the limitations and problems that come with the use of distance methods for phylogenetic inference. For example, Bromham argues,

[distance methods] tend to return an incorrect phylogeny under several common scenarios (for example when rates of molecular evolution vary between lineages […]). […] A distance tree is just a way of displaying information about similarities and differences. It may reflect evolutionary relationships, because descent with modification tends to leave a hierarchical pattern of differences. But just because we can draw a tree from a distance matrix does not mean we have uncovered evolutionary history. (Bromham 2016, p. 347)

While the term “phenogram” is usually absent from the glossary of molecular phylogenetics textbooks, the term “cladogram” was retained. As cladograms had already been used to represent evolutionary relatedness, they could easily be transformed into molecular phylogenetic trees. In the context of molecular phylogenetics, cladograms are usually interpreted as phylogenetic trees without information on branch lengths (Knoop and Müller 2009). Cladograms can thus be used to determine monophyletic groups, but they do not provide information on the number of evolutionary changes within a lineage (Lemey et al. 2009).

The distinction between phylogenetic trees and cladograms as trees with and without specified ancestors, respectively, that had been emphasized by some cladists in the late 1970s and early 1980s, has become obsolete, because today neither cladograms nor other types of molecular phylogenetic trees contain specified ancestors. In phylogenetic analysis all recent taxa within a group are treated as sister taxa that are represented by the external branches (Baum and Smith 2012). In this sense, molecular phylogenetics is still rooted in the cladistic approach advocated by Hennig. The internal and usually unnamed nodes of phylogenetic trees can be interpreted as actual or hypothetical common ancestors, speciation events, and/or the emergence of unique derived characters.^25^ Although the debate of the difference between cladograms and phylogenetic trees has largely subsided, the biological meaning of tree diagrams used in systematics and evolutionary biology remains unclear (Martin et al. 2010).

Maximum parsimony as the central cladistic method of tree inference has also remained an important part of the molecular phylogenetics toolkit. Although parsimony algorithms originated in pre-molecular systematics and were originally developed to construct cladograms from morphological characters, they can also be applied to molecular data by estimating the minimum number of nucleotide substitutions (Nei and Kumar 2000, pp. 115ff). With the integration of parsimony methods into a molecular framework and the shift of emphasis from classification to phylogenetic analysis, the use of the term “cladistics” had changed. David Williams and collaborators argue, “[i]nitially, cladistics was equated with Hennigian phylogenetic systematics. Later, the term ‘cladistics’ was used to refer to the application of parsimony algorithms in systematics” (Williams et al. 2010, p. 174).

Today, parsimony-based approaches are often perceived as outdated and inferior to so-called model-based approaches such as Maximum Likelihood. Some researchers view Maximum Parsimony merely as “a useful ‘fallback’ method when model-based methods cannot be used due to computational limitations” (Swoffort and Sullivan 2009, p. 269). Many molecular phylogeneticists prefer Maximum Likelihood methods over parsimony approaches because they are based on an explicit model of evolution. However, the debate between proponents of likelihood and other model-based statistical approaches and those who favor parsimony approaches is still unsettled.

## Conclusions

My analysis of the integration of systematics and molecular evolution, which gave rise to the field of molecular phylogenetics, shows that focusing on the conflicts between evolutionary systematists, numerical taxonomists and cladists neglects the force of a broader transformation of biological research. Automatization, mathematization, evolutionization, and quantification reshaped systematics profoundly and overshadowed the debate that revolved around theories and practices of classification. Technological advancements eventually led to the automation of sequencing and the introduction of cheap personal computers into systematics, which promoted the molecularization of phylogenetics and initiated a new era (Hughes 1999).

The construction of phylogenetic trees by using statistical methods was initiated largely independently in systematics and molecular evolution. These practices were integrated and further developed to eventually dominate molecular phylogenetics. It would thus be mistaken to portray either cladists or numerical taxonomists as victors of the Systematist Wars. However, it was the case that numerical taxonomists like Robert Sokal played an important role in developing computational techniques for phylogenetic analysis, although most numerical taxonomists viewed phylogenetic inference as a highly speculative endeavor. While it is true that some elements of Hennigian theory persisted and parsimony algorithms are still used for phylogenetic analysis, molecular phylogenetics is not a direct descendent of cladistics, but emerged independent of theories in systematics. The eventual integration of practices from systematics with practices of molecular evolution was possible, because they fit into the prevailing quantitative framework. With the molecularization of systematics and the shift of emphasis from classification to phylogenetic analysis, cladistics and phenetics are no longer perceived as different theoretical frameworks, but rather as different methods of studying molecular evolution (Williams et al. 2010). My study thus indicates that this episode of scientific change is more complex than common narratives of battles or compromises in systematics suggest.

In the context of molecular phylogenetics, phenograms were reinterpreted as molecular phylogenetic trees, and cladograms became molecular phylogenetic trees that do not represent the amount of evolutionary change within lineages. With the integration of practices of systematics with molecular evolution, phenograms and cladograms are no longer used to represent different forms of knowledge. Instead, both UPGMA-based trees and cladograms are now used to represent evolutionary relationships between taxa. This suggests that diagrams are versatile and somewhat flexible elements of scientific practice that can change their meaning, depending on the context of use within theoretical frameworks.

## Footnotes

1 See especially Chap. 5; see also Vernon (1988), Felsenstein (2001), Rieppel (2007) and Sterner and Lidgard (2018).

2 See especially Chap. 1 of Brower and Schuh (2021).

3 See for example, Sterner and Lidgard (2018); and see Kendig and Witteveen (2020).

4 See also Mayr (1996).

5 See Sneath and Sokal (1973), especially Chap. 5.9.

6 For more see Wiley (1981) p. 98.

7 In this article I use the term “evolutionary relationships” to refer to ancestor-descendent relationships or relationships based on common ancestry (e.g. sister taxa).

8 See for example, Cracraft (1979), Harper (1976), Platnick (1977).

9 Wiley defines a cladogram as “a branching diagram of entities where the branching is based on inferred historical connections between the entities as evidenced by synapomorphies” (Wiley 1981, p. 97).

10 An apomorphic character state is a derived character state unique to a group of species that has not been present in the ancestor and a plesiomorphic character state is an ancestral character state.

11 See Eldredge and Cracraft (1980, p. 67).

12 See Farris (1970).

13 See Suárez-Díaz and Anaya-Muñoz (2008).

14 See also Vernon (1988) and Vernon (1993).

15 See also Vernon (1988), particularly page 144.

16 See also Kluge and Farris (1969).

17 For a detailed account of the history of the use of molecular data in phylogenetic analysis see Suárez-Díaz (2014).

18 See also Strasser (2010) and Suárez-Díaz (2014).

19 This does not mean that molecular evolutionists operated completely separately from systematics. For example, some molecular evolutionists engaged with methods developed in systematics such as Wagner tree (Fitch 1971).

20 See also Edwards and Cavalli-Sforza (1964).

21 See Baverstock et al. (1979).

22 See Hagen (2001) and Suárez-Díaz (2014).

23 See Hennig (1996, p. 76).

24 See for example, Bromham (2016), Knoop and Müller (2009), Nei and Kumar (2000), Wiley and Lieberman (2011).

25 See Maddison and Maddison (2000, p. 37ff) for different interpretations of phylogenetic trees.

## References

[CR1] Atchley, William R., 2011. Walter M. Fitch (1929–2011). *Science* 332: 804. 10.1126/science.1207426. 10.1126/science.1207426 21566183

[CR2] Baum, David A., and Stacey D. Smith. 2012. *Tree thinking: An introduction to phylogenetic biology*. Greenwood Village: Roberts & Company.

[CR3] Baverstock, Peter R., Stephen R. Cole, Barry J. Richardson, and Christopher H. S. Watts. 1979. Electrophoresis and cladistics. *Systematic Zoology* 28: 214–219. 10.2307/2412524.

[CR4] Bromham, Lindell. 2016. *An introduction to molecular evolution and phylogenetics*. 2nd ed. Oxford, New York: Oxford University Press.

[CR5] Brower, Andrew V. Z., and Randall T. Schuh. 2021. *Biological systematics: Principles and applications*. 3rd ed. Ithaca: Cornell University Press.

[CR6] Camin, Joseph H., and Robert R. Sokal. 1965. A method for deducing branching sequences in phylogeny. *Evolution* 19: 311–326. 10.2307/2406441.

[CR7] Cavalli-Sforza, Luigi L., Anthony, and W. F. Edwards. 1967. Phylogenetic analysis. Models and estimation procedures. *American Journal of Human Genetics* 19: 233–257. 10.2307/2406616. PMC1706274 6026583

[CR8] Cracraft, Joel. 1979. Phylogenetic analysis, evolutionary models, and paleontology. In *Phylogenetic analysis and paleontology*, ed. Joel Cracraft, and Niles Eldredge, 7–40. New York: Columbia University. 10.7312/crac92306-003

[CR9] Edwards, Anthony W. F., and Luigi L. Cavalli-Sforza. 1964. Reconstruction of evolutionary trees. In *Phenetic and phylogenetic classification*, ed. Vernon H. Heywood and John McNeill, 67–76. London: Systematics Association.

[CR10] Eldredge, Niles and Joel Cracraft. 1980. *Phylogenetic patterns and the evolutionary process: Method and theory in comparative biology*. New York: Columbia University.

[CR11] Farris, James S. 1970. Methods for computing Wagner trees. *Systematic Zoology* 19: 83–92. 10.2307/2412028.

[CR12] Farris, James S., Arnold G. Kluge, and Michael J. Eckardt. 1970. A numerical approach to phylogenetic systematics. *Systematic Zoology* 19: 172–189. 10.2307/2412452.

[CR13] Felsenstein, Joseph. 2001. The troubled growth of statistical phylogenetics. *Systematic Biology* 50: 465–467. 10.1080/10635150119297. 12116645 10.1080/10635150119297

[CR15] Fitch, Walter M. 1971. Toward defining the course of evolution: Minimum change for a specific tree topology. *Systematic Biology* 20: 406–416. 10.1093/sysbio/20.4.406.

[CR16] García-Sancho, Miguel. 2012. *Biology, computing, and the history of molecular sequencing*. New York: Palgrave Macmillan.

[CR17] Goodman, Nelson. 1976. *Languages of art. An approach to a theory of symbols*. Indianapolis: Hackett Publishing Company.

[CR18] Gould, Stephen Jay. 1995. Ladders and cones: Constraining evolution by canonical icons. In *Hidden histories of science*, ed. R. Silvers, 37–68. New York: New York Review Books.

[CR21] Hagen, Joel B. 2001. The introduction of computers into systematic research in the United States during the 1960s. *Studies in History and Philosophy of Science Part C: Studies*. *In History and Philosophy of Biological and Biomedical Sciences* 32: 291–314. 10.1016/S1369-8486(01)00005-X.

[CR22] Harper, Charles W. 1976. Phylogenetic inference in paleontology. *Journal of Paleontology* 50: 180–193.

[CR23] Hennig, W. 1966. *Phylogenetic systematics*. Urbana,Illinois: University of Illinois Press.

[CR24] Hughes, Austin L. 1999. *Adaptive evolution of genes and genomes*. Oxford: Oxford University Press.

[CR25] Hull, David L. 1988. *Science as a process: An evolutionary account of the social and conceptual development of science*. Chicago: University of Chicago Press.

[CR26] Huxley, Julian. 1940. *The new systematics*. Oxford: Clarendon.

[CR27] Kendig, Catherine, and Joeri Witteveen. 2020. The history and philosophy of taxonomy as an information science. *History and Philosophy of the Life Sciences* 42: 1–9. 10.1007/s40656-020-00337-8. 10.1007/s40656-020-00337-8 32865722

[CR28] Kluge, Arnold G., and James S. Farris. 1969. Quantitative phyletics and the evolution of anurans. *Systematic Biology* 18: 1–32. 10.1093/sysbio/18.1.1.

[CR29] Knoop, Volker and Kai Müller. 2009. *Gene und Stammbäume*. Heidelberg: Spektrum Akademischer.

[CR30] Lemey, Philippe, Marco Salemi, and Anne-Mieke Vandamme. eds. 2009. *The phylogenetic handbook: A practical approach to phylogenetic analysis and hypothesis testing*. 2nd edn. Cambridge: Cambridge University Press.

[CR31] Maddison, David R., and Wayne P. Maddison. 2000. *MacClade 4*. Sunderland: Sinauer.

[CR32] Martin, Jeremy, David Blackburn, and Edward O. Wiley. 2010. Are node-based and stem-based clades equivalent? Insights from graph theory. *PLOS Currents Tree of Life*, November. 10.1371/currents.RRN1196. 10.1371/currents.RRN1196 PMC2989695 21113336

[CR33] Mayr, Ernst. 1942. *Systematics and the origin of species from the viewpoint of a zoologist*. 1st ed. New York: Columbia University.

[CR34] Mayr, Ernst. 1969. *Principles of systematic zoology*. New York: McGraw-Hill.

[CR35] Mayr, Ernst. 1996. What is a species, and what is not? *Philosophy of Science* 63: 262–277.

[CR36] Nei, Masatoshi, and Sudhir Kumar. 2000. *Molecular evolution and phylogenetics*. Oxford: Oxford University Press.

[CR37] Platnick, Norman I. 1977. Cladograms, phylogenetic trees, and hypothesis testing. *Systematic Zoology* 26: 438–442. 10.2307/2412799.

[CR38] Ragan, Mark A. 2009. Trees and networks before and after Darwin. *Biology Direct* 4: 43. 10.1186/1745-6150-4-43. 19917100 10.1186/1745-6150-4-43 PMC2793248

[CR39] Rieppel, Olivier. 2007. The metaphysics of Hennig’s phylogenetic systematics: Substance, events and laws of nature. *Systematics and Biodiversity* 5: 345–360. 10.1017/S1477200007002575.

[CR40] Schnell, Gary D. 1970. A phenetic study of the suborder Lari (Aves). II. Phenograms, discussion, and conclusions. *Systematic Zoology* 19: 264–302. 10.2307/2412211. 5466091

[CR42] Simpson, George G. 1961. *Horses. The story of the horse family in the modern world and through sixty million years of history*. New York: Anchor Books.

[CR44] Sneath, Peter H. A., and Robert R. Sokal. 1973. *Numerical taxonomy. The principles and practice of numerical classification*. San Francisco: W. H. Freeman and Company.

[CR45] Sokal, Robert R, and Peter H. A. Sneath. 1963. *Principles of numerical taxonomy*. San Francisco: W. H. Freeman and Company.

[CR46] Spicer, Greg S. 1985. A new fairy shrimp of the genus Streptocephalus from Mexico with a phylogenetic analysis of the north American species (Anostraca). *Journal of Crustacean Biology* 5: 168–174. 10.2307/1548229.

[CR47] Sterner, Beckett, and Scott Lidgard. 2014. The normative structure of mathematization in systematic biology. *Studies in History and Philosophy of Science Part C: Studies in**History and Philosophy of Biological and Biomedical Sciences* 46: 44–54. 10.1016/j.shpsc.2014.03.001 10.1016/j.shpsc.2014.03.001 24717645

[CR48] Sterner, Beckett, and Scott Lidgard. 2018. Moving past the systematics wars. *Journal of the History of Biology* 51: 31–67. 10.1007/s10739-017-9471-1 28255641 10.1007/s10739-017-9471-1

[CR49] Strasser, Bruno J. 2010. Collecting, comparing, and computing sequences: The making of Margaret O. Dayhoff’s ‘Atlas of protein sequence and structure,’ 1954–1965. *Journal of the History of Biology* 43: 623–660. 10.1007/s10739-009-9221-0 20665074 10.1007/s10739-009-9221-0

[CR50] Suárez-Díaz, Edna. 2009. Molecular evolution: Concepts and the origin of disciplines. *Studies in History and Philosophy of Science Part C: Studies in**History and Philosophy of Biological and Biomedical Sciences* 40: 43–53. 10.1016/j.shpsc.2008.12.006 10.1016/j.shpsc.2008.12.006 19268873

[CR51] Suárez-Díaz, Edna. 2014. The long and winding road of molecular data in phylogenetic analysis. *Journal of the History of Biology* 47: 443–478. 10.1007/s10739-013-9373-9. 25574534

[CR52] Suárez-Díaz, Edna, and Victor Anaya-Muñoz. 2008. History, objectivity, and the construction of molecular phylogenies. *Studies in History and Philosophy of Science Part C: Studies in History and Philosophy of Biological and Biomedical Sciences* 39: 451–468. 10.1016/j.shpsc.2008.09.002. 10.1016/j.shpsc.2008.09.002 19026976

[CR53] Swofford, David L., and Jack Sullivan. 2009. Phylogeny inference based on parsimony and other methods using PAUP. In *The phylogenetic handbook: A practical approach to phylogenetic analysis and hypothesis testing*, ed. Anne-Mieke Vandamme, Marco Salemi and Philippe Lemey, 2nd edn., 267–312. Cambridge: Cambridge University Press.

[CR54] Van de Peer, Yves. and Marco Salemi. 2009. Phylogenetic inference based on distance methods. In *The Phylogenetic handbook: A practical approach to Phylogenetic analysis and hypothesis testing*, ed. Anne-Mieke Vandamme, Marco Salemi and Philippe Lemey, 2nd edn., 142–180. Cambridge: Cambridge University Press.

[CR55] Vandamme, Anne-Mieke. 2009. Basic concepts of molecular evolution. In *The phylogenetic handbook: A practical approach to phylogenetic analysis and hypothesis testing*, ed. Anne-Mieke Vandamme, Marco Salemi and Philippe Lemey, 2nd edn., 3–30. Cambridge: Cambridge University Press.

[CR56] Vernon, Keith. 1988. The founding of numerical taxonomy. *The British Journal for the History of Science* 21: 143–159. 10.1017/S0007087400024730.

[CR57] Vernon, Keith. 1993. Desperately seeking status: Evolutionary systematics and the taxonomists’ search for respectability 1940-60. *The British Journal for the History of Science* 26: 207–227. 10.1017/S0007087400030764.

[CR58] Vorms, Marion. 2011. Formats of representation in scientific theorizing. In *Models, simulations, and representations*, ed. Paul Humphreys and Cyrille Imbert, 250– 73. New York: Routledge.

[CR59] Wiley, Edward O. 1981. *Phylogenetics: The theory and practice of phylogenetic systematics*. New York: Wiley.

[CR60] Wiley, Edward O., and Bruce S. Lieberman. 2011. *Phylogenetics. Theory and practice of phylogenetic systematics*. 2nd edn. Hoboken: Wiley-Blackwell.

[CR61] Williams, David M., Malte C., Ebach, and Quentin D. Wheeler. 2010. Beyond belief. The steady resurrection of phenetics. In *Beyond cladistics. The branching of a paradigm*, ed. David M. Williams, and Sandra Knapp. 169– 95. Berkeley: University of California Press.

